# Comparison of Ventricular and Lumbar Cerebrospinal Fluid Composition

**DOI:** 10.7759/cureus.9315

**Published:** 2020-07-21

**Authors:** Stacey Podkovik, Samir Kashyap, James Wiginton, Christine Kang, Kevin Mo, Mackenzie Goodrich, Adam Wolberg, Margaret Rose Wacker, Dan E Miulli

**Affiliations:** 1 Neurosurgery, Riverside University Health System Medical Center, Moreno Valley, USA; 2 Neurosurgery, Touro University College of Osteopathic Medicine California, Vallejo, USA; 3 Medicine, Western University of Health Sciences, Pomona, USA; 4 Neurosurgery, Burrell College of Osteopathic Medicine, Las Cruces, USA; 5 Surgery, Lake Erie College of Osteopathic Medicine, Bradenton, USA; 6 Neurosurgery, Arrowhead Regional Medical Center, Colton, USA

**Keywords:** cerebrospinal fluid, lumbar drain, lumbar puncture, external ventricular drain, infection, csf composition, csf

## Abstract

Objective

Cerebrospinal fluid (CSF) analysis is a common diagnostic tool used to evaluate diseases of the central nervous system (CNS). We sought to determine whether there is a difference between the composition of CSF sampled from an external ventricular drain (EVD) and lumbar drain (LD) and whether this made a difference in guiding therapeutic decisions.

Patients and Methods

This study was a retrospective analysis from a single neurosurgery service between the dates of January 2011 and April 2019. A total of 12,134 patients were screened. Inclusion criteria were ages 18-80 and the presence of both an EVD and LD. Exclusion criteria were not having both routes of CSF sampling and the inability to determine which samples originated from which compartment.

Results

Six patients underwent simultaneous spinal and ventricular routine CSF sampling <24 hours apart and were analyzed for their compositions. There were 42 samples, but only 20 paired EVD-LD samples that could be analyzed. When comparing the EVD and LD sample compositions, there were statistically significant differences in white blood cells (WBCs; p = 0.040), total protein (p = 0.042), and glucose (p = 0.043). Red blood cells (RBCs; p = 0.104) and polymorphonuclear leukocytes (PMN; p = 0.544) were not statistically significant. We found a statistically significant correlation between cranial and spinal CSF WBC (r = 0.944, p < 0.001), protein (r = 0.679, p = 0.001), and glucose (r = 0.805, p < 0.001). We also found that there was a significant correlation between CSF and serum glucose (r = 0.502, p = 0.040). There was no statistically significant correlation between RBCs (r = 0.276, p = 0.252).

Conclusion

Our results demonstrate a correlation between the cranial and spinal CSF samples, except for RBCs, with statistically significant differences in WBC, glucose, and protein values between the two sites. This confirms that sampling CSF via lumbar puncture, which carries less risk than a ventriculostomy and provides accurate data to help establish a diagnosis for intracranial pathologies.

## Introduction

Analysis of cerebrospinal fluid (CSF) is a common diagnostic tool used to evaluate various pathologies of the central nervous system (CNS). CSF sampling requires performing invasive procedures such as lumbar puncture, lumbar drain (LD), and ventriculostomy placement that can introduce the patient to many potential complications, including infections such as meningitis [1].

Controversy exists as to whether there is a consistent significant difference between compositions of CSF in the ventricular system compared to the spinal compartment that is sampled via lumbar puncture. A review of the literature shows that, particularly in infectious processes, there may exist a discrepancy between ventricular and lumbar CSF sample composition. On the other hand, for some pathologies, cases illustrating that there is no statistically significant difference between the CSF composition of malignant cells in these two regions have similarly been reported [2]. This raises the question of whether the location from which CSF was obtained is of clinical and diagnostic importance [3].

In this study, we sought to determine whether there is a significant difference between the composition of CSF sampled from an external ventricular drain (EVD) and LD and whether this made a difference in guiding therapeutic decisions.

## Materials and methods

This study was a retrospective analysis of a prospectively collected database from a single neurosurgery service between the dates of January 2011 and April 2019. A total of 12,134 patients were screened from our institution’s neurosurgical database based on a query for “EVD & LD.” Twelve patients were included in this study (nine males and three females) with ages ranging from 23 to 65 years old. Inclusion criteria were patients ages 18-80 and the simultaneous presence of both an EVD and LD. Exclusion criteria were not having both routes of CSF sampling in place at the same time and the inability to determine which samples originated from which compartment. We also excluded individuals who had samples drawn within 24 hours of any surgical intervention.

The placement of both the EVD and LD was performed at the bedside or in the operating room using standard sterile technique. Ventricular and lumbar CSF were collected through their respective sampling ports of the catheter either at the bedside or in the operating room and tested within four hours of collection. Samples were collected simultaneously to compare the composition of white blood cells (WBCs), red blood cells (RBCs), protein, glucose, lactate (when available), and polymorphonuclear leukocyte (PMN) percentages from both the EVD and LD CSF. We also collected infectious markers such as Gram stain and culture for the CSF.

All data analysis was completed using the Statistical Package for Social Sciences (SPSS, IBM Version 23, SPSS Incorporation, Chicago, USA). Paired sample T-tests were utilized for assessing differences between EVD and LD group means, and Pearson correlations were used to determine any relationship between integral variables. A statistical significance level of less than 5% was used.

## Results

Twelve patients were found who had a simultaneous EVD and LD in place. Seven patients had intracranial hemorrhage, three had an infection, one presented with a tumor, and one had a vascular malformation. A total of six patients (five male and one female) underwent spinal and ventricular routine CSF sampling <24 hours apart, for prophylactic purposes, and were analyzed for their compositions. Of the six patients who met all inclusion criteria, there were 42 total CSF analyses, but only 20 paired EVD-LD samples that could be used for analysis. The average ages were 40 years of age for the female and 40.33 ± 13.08 years for males (p = 0.975). When comparing the EVD and LD sample compositions, there were statistically significant differences in WBC (p = 0.040), total protein (p = 0.042), and glucose (p = 0.043). Differences in RBC (p = 0.104) and PMN (p = 0.544) were not statistically significant. Of the six patients, who were included within the study, four individuals had at least one positive culture (two were Klebsiella pneumoniae, one was vancomycin-resistant Enterococcus faecium, and one was methicillin-resistant Staphylococcus aureus (MRSA) plus Escherichia coli). Only one of the four patients who had a positive culture had positive cultures within two simultaneous EVD and LD samples. This positive culture grew K. pneumoniae. The individual patient lab values can be found in (Table 1).

**Table 1:** Cerebrospinal fluid profiles EVD: external ventricular drain, LD: lumbar drain, WBC: white blood cells, RBC: red blood cells, PMN: polymorphonuclear leukocyte.

Patient Number	Gender	Pathology	Sample Number	EVD WBC	EVD RBC	EVD Protein	EVD Glucose	EVD PMN %	LD WBC	LD RBC	LD Protein	LD Glucose	LD PMN %
1	Male	Infection	1	5	1000	109	53	20	49	1000	519	32	2
			2	8	3000	92	55	38	62	3000	594	32	3
2	Male	ICH	1	25806	3000	1353	2	97	40764	1000	3630	<2	95
3	Male	ICH	1	5	1000	41	41	40	3	2	<4	50	1
			2	0	0	31	53	-	1	0	30	42	100
4	Male	Tumor	1		150	427	56	55	12	100	1692	25	80
			2	8	30	142	82	72	93	550	990	85	69
			3	2	0	29	81	-	9	4	30	84	8
5	Female	ICH	1	2	0	24	5	-	9016	1000	84	21	84
			2	7	30	14	51	-	7	35	32	24	37
			3	2	0	10	45	-	132	1000	26	22	2
			4	-	-	10	62	-	-	-	4	55	-
			5	0	0	8	38	-	5	5	23	38	-
			6	0	0	8	43	0	2	0	21	41	-
6	Male	ICH	1	13		140	115	95	6930	426000	142	106	83
			2	166	5000	54	125	5	9730	140000	146	120	95
			3	15	50	30	113	20	1005	205000	75	88	72
			4	25	28000	15	72	24	24	80000	65	76	46
			5	0	2	12	89	-	736	19000	56	83	71
			6	10	100	60	65	30	0	1	53	82	-

We found a statistically significant linear correlation between cranial and spinal CSF WBC (r = 0.944, p < 0.001), protein (r = 0.679, p = 0.001), and glucose (r = 0.805, p < 0.001). We also found that there was a significant correlation between CSF and serum glucose (r = 0.502, p = 0.040). There was no statistically significant correlation between RBCs (r = 0.276, p = 0.252).

## Discussion

Our study aimed to determine whether there is a significant difference between the composition of cranial and spinal CSF by simultaneously sampling CSF from both an EVD and LD catheter under standardized conditions. This is particularly relevant in patients with a suspected CNS infection. In contrast to previous studies by Kakadia et al. and Sommer et al. which evaluated for any differences between EVD and lumbar puncture, we sought to use an LD in order to allow us to do serial sampling at various times in each patient to evaluate for temporal differences [4,5]. CSF is steadily produced, circulated, and reabsorbed within the CNS at a rate of approximately 20cc per hour [6-8]. Given the connected, steady-state of CSF production and reabsorption, the question arises whether sampling lumbar spinal fluid would yield sufficiently accurate diagnostic information in the presence of a CNS infection. Kakadia et al. reviewed a series of six cases of bacterial meningitis and postulated that the differences between the WBC counts from EVD and lumbar puncture sources may have been due to slower circulation of CSF within the lumbar compartment [4]. Rubalcava and Sotelo demonstrated that CSF protein increases as it moves along the typical pathway from the ventricles to the spine and back to the subarachnoid villi [9].

Our results demonstrated significant differences in WBC, protein, and glucose between cranial and spinal CSF samples. The trends appear to show a larger concentration of WBC and protein within the lumbar compartment, whereas glucose tends to be higher within the ventricles. Our results demonstrate that sampling the lumbar spinal fluid would likely over-estimate the presence of true infections, but remains an overall safer diagnostic modality compared to ventriculostomies. Additionally, there was a clear correlation between cranial and spinal CSF WBC, protein, and glucose counts. One potential explanation for the lack of RBC correlation is that LD or puncture procedures frequently can elicit “traumatic taps,” which occur in 10-30% of these procedures [10]. This is in contrast to the 14.8-21.6% hemorrhage rates following EVD placement [11,12]. This can lead to significantly elevated numbers of RBCs in the lumbar cistern as compared to the ventricular one. The WBCs do not appear to show this similar trend because there has been ample documentation of an RBC:WBC ratio in CSF of about 500-1000:1, indicating that the proportional difference would likely be too small to yield a statistically significant difference [13,14].

Similarly, we found a moderate correlation between CSF and serum glucose values. This is an expected finding, as studies such as Hegen et al. demonstrated that CSF glucose is typically about 60% of the serum glucose levels [15]. Of particular interest is why three of the four patients with positive CSF cultures only demonstrated infection in either the ventricular or lumbar cistern, but not both. The three individuals had one lumbar culture of K. pneumoniae, one ventricular culture of E. faecium, and one ventricular culture of MRSA and E. coli. In all four culture-positive patients, the Gram stains all resulted as negative. A possible explanation as to why cultures in only one compartment or the other resulted as positive could be due to the slow seeding of bacteria from the cranial to spinal regions. This is clinically important because it demonstrates that when someone has an intracranial infection, there is a possibility that the lumbar spinal fluid cultures may not yield a particular bacterial species. Another more likely explanation for this finding is that these positive cultures are simply a contaminant, especially in one sample that grew two separate organisms. We believe that the positive CSF culture from both drains represented a true infection.

The retrospective nature of this study introduces an inherent selection bias. It may also contribute to inconsistent analysis for serum protein and lactate levels, but the CSF parameters can be utilized to inform a prospective study. A second limitation of this study was the low sample size. Unfortunately, many samples were unable to be incorporated into statistical analysis because they were inadequately labeled within the electronic medical record, making it impossible to determine which samples were ventricular versus lumbar.

## Conclusions

CSF sampling comes with inherent risks to patients, but is necessary for diagnostic and therapeutic purposes. Our results demonstrate a strong correlation between the cranial and spinal CSF samples, except for RBCs, with statistically significant differences in WBC, glucose, and protein values between the two compartments. Despite the differences between ventricular and spinal compartments, these CSF components trend similarly. It further affirms that the practice of sampling CSF via lumbar puncture, which carries significantly less risk than a ventriculostomy, provides accurate trends to help establish a diagnosis for intracranial pathologies.
